# Remarkable Population Resilience in a North African Endemic Damselfly in the Face of Rapid Agricultural Transformation

**DOI:** 10.3390/insects12040353

**Published:** 2021-04-15

**Authors:** Rassim Khelifa, Hayat Mahdjoub, Affef Baaloudj, Robert A. Cannings, Michael J. Samways

**Affiliations:** 1 Department of Zoology, University of British Columbia, Vancouver, BC V6T 1Z4, Canada; 2 Biodiversity Research Centre, University of British Columbia, Vancouver, BC V6T 1Z4, Canada; 3 Department of Evolutionary Biology and Environmental Studies, University of Zurich, Winterthurerstrasse 190, 8057 Zurich, Switzerland; hayatmahdjoub@gmail.com; 4 Laboratoire Biologie, Eau & Environnement (LBEE), Faculty of SNV-STU, University of 8 May 1945, Guelma 24000, Algeria; bafef@yahoo.fr; 5 Entomology, Royal British Columbia Museum, 675 Belleville Street, Victoria, BC V8W 9W2, Canada; rcannings@royalbcmuseum.bc.ca; 6 Department of Conservation Ecology and Entomology, Stellenbosch University, Victoria Street, Stellenbosch 7602, South Africa; samways@sun.ac.za

**Keywords:** Odonata, Zygoptera, agroecosystems, habitat degradation, aquatic insects, river

## Abstract

**Simple Summary:**

There has been a rapid expansion of agricultural area worldwide, resulting in a substantial change in the physical structure, biodiversity, and ecosystem functioning of various natural habitats. In North Africa, many natural habitats have been transformed into agricultural lands, especially in the North, where biodiversity is the highest, to meet the food security and economic development of a rapidly growing population. We estimated the agricultural expansion in North African region between the 1990s and 2000s and found that the percentage of agriculture-free area within the species range declined from 79.5% to 26.2%. Knowing that agroecosystems near lotic environments simplify the structural complexity of habitats (from heterogeneous to homogenous ecological communities) of amphibiotic species such as odonates, we estimated the geographic range of an endemic damselfly and quantified the temporal change in the overlap between agriculture and species occurrence. Our results showed the overlap more than tripled between 1992 and 2005, suggesting that the species experienced a radical change in its terrestrial habitats. We conducted capture–mark–recapture to confirm that the species survives by frequently using croplands and grasslands.

**Abstract:**

Agriculture can be pervasive in its effect on wild nature, affecting various types of natural habitats, including lotic ecosystems. Here, we assess the extent of agricultural expansion on lotic systems in Northern Africa (Algeria, Tunisia, and Morocco) and document its overlap with the distribution of an endemic damselfly, *Platycnemis subdilatata* Selys, using species distribution modeling. We found that agricultural land cover increased by 321% in the region between 1992 and 2005, and, in particular, the main watercourses experienced an increase in agricultural land cover from 21.4% in 1992 to 78.1% in 2005, together with an increase in the intensity of 226% in agricultural practices. We used capture–mark–recapture (CMR) surveys in terrestrial habitats surrounding a stream bordered by grassland and cropland in northeastern Algeria to determine demographic parameters and population size, as well as cropland occupancy. CMR modeling showed that the recapture and survival probabilities had an average of 0.14 (95%CI: 0.14–0.17) and 0.86 (0.85–0.87), respectively. We estimated a relatively large population of *P. subdilatata* (~1750 individuals) in terrestrial habitats. The occupancy of terrestrial habitats by adults was spatially structured by age. Our data suggest that *P. subdilatata* has survived agricultural expansion and intensification better than other local odonate species, mainly because it can occupy transformed landscapes, such as croplands and grasslands.

## 1. Introduction

Much of the global wilderness has been converted to agricultural lands to feed humans and livestock [1,2]. Consequently, many natural ecosystems are now fragmented by agroecosystems, interfering with important ecological processes [3]. Rivers, for instance, are commonly bordered by croplands, shifting the natural environment from diverse native communities of plants and animals to more simplified agroecosystems with reduced biotic diversity [4,5]. Understanding the extent of agroecosystems across riparian ecosystems and how they affect occupancy by freshwater organisms is crucial for establishing mitigation measures for the conservation of biodiversity.

In North Africa, agriculture is mainly localized near the coast, where most fertile lands and permanent waterbodies occur [6]. The ecological barriers around this fertile region (the Sahara to the south) naturally restrict the most biologically diverse communities and human population to the coastal area [7]. Following rapid human population growth in the region in the last few decades [8], the agricultural area has expanded greatly, and farming practices have shifted to intensive monoculture systems to meet food security and economic growth [9]. This extensive and intensive conversion of natural habitats has most likely affected local lotic biodiversity in various ways. Lotic systems are commonly used for irrigation through water abstraction, potentially killing aquatic organisms by transferring them to terrestrial habitats, reducing water levels, and changing the physicochemical properties of the water and the ecological interactions among organisms and communities [10]. Furthermore, use of pesticides contaminates the water with toxic compounds and exposes aquatic organisms, such as insect larvae, to water pollution [11], leading to reduction in biodiversity both in the water and along the riparian zone [12,13,14]. In addition, extensive use of fertilizers often leads to heavy metal contamination and eutrophication of the water, with substantial repercussions on the diversity and composition of aquatic fauna [15,16]. Therefore, an understanding of the extent of agricultural expansion across lotic ecosystems is crucial for predicting current and future impacts on the abundance and composition of biodiversity.

Among freshwater insects with complex life cycles, such as odonates (Odonata: Anisoptera and Zygoptera), adults frequently use terrestrial habitats near the aquatic reproductive sites [17]. After emergence, the adults of temperate damselflies generally spend a few days to weeks in terrestrial habitats foraging and gaining mass to reach maturity. Once mature, they also use terrestrial habitats for foraging. The diet of dragonflies and damselflies is diverse [18,19], including mostly flying insects from various orders (Diptera, Hymenoptera, Hemiptera, etc.). This suggests that an environmental shift from native natural plant communities to monospecific agroecosystems changes the quantity and quality of the prey for dragonflies, and could ultimately affect their individual fitness and population dynamics [20,21].

Most North African lotic species are listed on the IUCN Red List as threatened [22,23]. For instance, the damselfly *Calopteryx exul* Selys is endangered, and the dragonfly *Gomphus lucasii* Selys is vulnerable [24,25]. The only lotic endemic damselfly in North Africa of least concern is *Platycnemis subdilatata* Selys (Platycnemididae). This species is widespread in the region and occurs in large populations, suggesting that it frequently occupies agricultural lands near lotic habitats. Assessing the habitat fragmentation at the scale of the species’ geographic range would reflect the general conditions of the lotic habitat. Despite its abundance, this damselfly has been poorly studied [26,27].

Here, we investigate to what extent North African lotic habitats associated with agroecosystems impact *P. subdilatata*, and whether the adults use these agroecosystems during their lifespan. We specifically used capture–mark–recapture (CMR) to determine demographic parameters and population size of the adults in terrestrial habitats near a stream in northeastern Algeria (Seybouse watershed). We also investigated the species’ spatial distribution in the riparian zone and nearby croplands using regular transects to reveal whether the species occupied both grasslands and croplands. We finally assessed the distribution of *P. subdilatata* using species distribution modelling and tested for the temporal expansion in the distribution of croplands around its lotic habitat.

## 2. Materials and Methods

### 2.1. Study Area

The distribution of *P. subdilatata* was investigated in North Africa in the three countries where it is known to occur (Algeria, Tunisia, and Morocco) [28]. North Africa is one of the driest and warmest areas in the world and probably the warmest in the western Palearctic. The climate in the North is typically Mediterranean with hot dry summers and humid cool winters. In the area where the species occurs (Figure S1), the average annual temperature is 17.1 °C (sd = 2.75, range = 3.11–23.17 °C) and annual precipitation is 343 mm (sd = 205.7, range = 38–1554 mm) (WorldClim 2.1) [29].

### 2.2. Demographic Parameter

**Figure 1:**
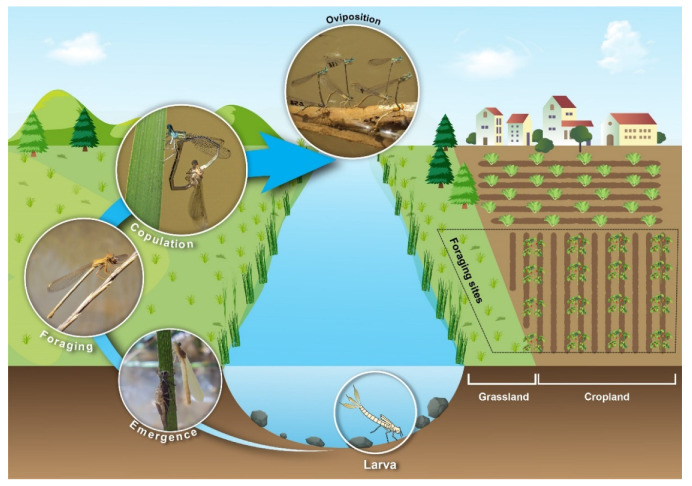
Typical life cycle of damselflies. The contoured area on the right is similar to the study site where the capture–mark–recapture study took place in northeastern Algeria (Bouhamra, Guelma). Although both sides of the studied watercourse had agricultural lands, we present here only one side (**right**) with agricultural modification and one side (**left**) without agriculture to highlight the historical change in land use.

We conducted capture–mark–recapture study in a shallow stream (36°27′24.50″ N, 7°30′29.53″ E) in a peri-urban area (Figure 1) about 6 km east of Guelma city, Boumahra, Northeast Algeria. The bank vegetation was dominated by *Typha angustifolia*, which is the most utilized substrate for oviposition of *P. subdilatata* [27]. *Salix pedicellata*, *Populus alba,* and *Eucalyptus globulus* trees bordered the watercourse. The riparian zone consisted of native grasslands dominated by stands of *Daucus carota* and *Cirsium vulgare*. Fields of tomato and lettuce were about 20 m away from the water, and their harvesting coincided with the appearance of adult *P. subdilatata* and other odonates.

Daily CMR was carried out by one person between May 6 and June 16, 2012 at 09:00 using transects across two patches of grassland and two patches of croplands, each 20 × 10 m^2^. Sampling was for 30 min in each of the two types of patches sequentially. Adults were captured with a hand net and marked on the left posterior wing with an alphanumeric code using permanent markers (fine Staedtler© permanent marker). Using prior information on adult coloration pattern [27] and softness of the body and wings, the age of individuals was determined by using three age classes: teneral (newly emerged < 24 h), immature (2–7 days), and mature individuals (>7 days). Due to their soft body and wings, tenerals were kept in cages in shaded areas and marked at the end of the afternoon (~17:00).

Cormack–Jolly–Seber (CJS) was used to estimate recapture (*p*) and survival (ψ) probabilities [30]. Ψ is the probability of surviving from occasion *i* to *i* + 1, and *p* is the probability of encountering a marked individual conditional on being alive. Capture histories, which consisted of a series of “0”s (not detected) and “1”s (detected), were generated for each individual. A capture history of 101, for instance, meant that the individual was marked and released on day 1, not recaptured on day 2, and observed again on day 3. The goodness-of-fit test (Test2, Test3, and the total of tests) on the CJS model was computed with the function *release.gof* implemented in the RMark package [31]. These tests focused on whether all marked individuals had an equal chance of being captured (Test2), and whether they had an equal probability of surviving (Test3) on any one occasion [32]. The variance inflation factor (c-hat), which is an estimate of the overdispersion that reflects how the model fits the observed data, was calculated using bootstrap resampling procedure with 1000 iterations, and model selection was adjusted accordingly.

The parameter estimates of *p* and Ψ were calculated using the R package RMark [31]. We built candidate models with increasing complexity from an intercept-only model for both parameters *p*(.) Ψ(.) to a more complex model: *p*(sex × time) Ψ(sex × age), which tests the additive and interactive effects of sex and time occasion on detectability, and sex and age on survival probability. Time occasion was tested as a continuous (Time) and a categorical (time) variable. Model selection was based on the AICc (corrected Akaike Information Criterion: ΔAICc ≥ 2 means a better model [33]) and parsimony. We used the average survival rate to estimate adult longevity (life expectancy) using the equation −1/log_e_(Ψ) [34].

To estimate the population size of the species, we used the POPAN model [35,36]. The *p* and Ψ were fixed to the best model found in CJS. Then, the “superpopulation” (total number of damselflies that ever entered the sampled area during the entire sampling period) was tested for the effect of sex [*N*(sex)], and the probability of entry (*pent*) into that superpopulation at each occasion was tested for the effect of sex and time [*pent*(sex + time)]. After model selection using AICc, we used the best model to predict the superpopulation size for the entire sampling period to obtain an overall estimate of the population size and for each sampling occasion to assess the seasonal pattern of abundance.

All statistics were performed using the software R 3.4.0 [37]. Chi-squared tests were carried out to test for differences in the proportion of males and females in foraging habitat. A negative binomial model was used to determine whether the spatial distribution of damselfly abundance in grassland and cropland across age classes was homogenous or structured. We tested for the main effect as well as three-way interaction of sex, age class, and land type (grassland or cropland) on the number of marked individuals per sampling occasion.

### 2.3. Agricultural Intensity Data

To determine the extent, intensity, and expansion of agricultural activity in North Africa, we used the cropland intensity index for 1992 and 2005 obtained from Venter et al. [38] with a resolution of 30 s (1 km^2^). Remotely sensed agricultural extent data were originally extracted from the UMD Land Cover Classification [39] and GlobCover [40]. This index varies from 0 to 7, with 0 indicating the absence of agricultural activity and 7 indicating intense activity. By comparing the cropland intensity index of 1992 and 2005, we estimated the change in the area of land occupied by croplands, as well as the change in intensity of agricultural activity.

### 2.4. Geographic Range Estimation

Presence–absence records of *P. subdilatata* were generated by analyzing a suite of studies and the GBIF (Global Biodiversity Information Facility) database on riverine odonates in North Africa (Algeria, Tunisia, and Morocco) (Table S1). Presence data were derived from actual observation of the species at a given site and absence data implied from odonatological surveys that did not record the presence of the species. *P. subdilatata* is the only *Platycnemis* in the region [28], and the species is easily distinguishable from other Zygoptera. The records used in this study were made by experienced odonatologists.

We linked the spatial distribution of *P. subdilatata* records to 19 bioclimatic variables (11 temperature and eight precipitation variables) and elevation available in WorldClim2.1 with 30 s resolution (~1 km^2^) [29]. To determine the species geographic distribution, we used the *sdm* R package [41] and tested for the association between the species occurrence and our predictors. After removing strong collinearity among predictors by applying a threshold of variance inflation factor (VIF) of 10 [42] using the function *vifstep* from the *usdm* package [43], we retained seven variables (BIO2, BIO3, BIO8, BIO13, BIO14, BIO18, and elevation) (Table S2). These variables reflect the thermal and rainfall conditions and likely play a role in determining the distribution of the species in the region. We used eleven commonly used models in species distribution models (SDM), namely, generalized linear models (GLM), generalized additive models (GAM), boosted regression trees (BRT), random forests (RF), multiple discriminant analysis (MDA), maximum entropy (Maxent), likelihood-based estimator adopted for presence-only data (Maxlike), multivariate adaptive regression splines (MARS), recursive partitioning and regression trees (RPART), flexible discriminant analysis (FDA), and BIOCLIM (based on the BIOCLIM package). We used the default parameterization option of *sdm* to compute the modeling.

For each method, we used the subsampling and bootstrapping methods, each replicated four times. We fitted ecological niche models for each replication. In subsampling procedure, 70% of data were used for training the model, and 30% of data were used for testing the model. Different metrics were used to evaluate the performance of model predictions, including the area under curve (AUC) of the receiver operating characteristic (ROC) plot and the true skill statistic (TSS) [44]. AUC varied from 0 to 1, where values near 1 reflected excellent model performance. TSS varied from −1 to 1, where 1 indicated perfect agreement. Then, we computed and projected the distribution of climate suitability for the species using the ensemble of all models across the North African region. We used AUC-weighted mean across all models to reach a consensus [45]. Figure S2 reports the model performance for each method. To infer the predictive power of each explanatory variable, we estimated the variable importance [46]. We selected a threshold for TSS that maximized both the true positive rate and true negative rate. Using this threshold and the ensemble predictions, we generated presence–absence data for *P. subdilatata* across the main watercourses in northern Africa. To determine the temporal change in extent of agriculture across the species range, 4 km^2^ hexagons were overlaid on the polyline of the lotic habitat where *P. subdilatata* was present, and then we calculated average agricultural intensity (obtained from Venter et al. [47]) within each hexagon for 1992 and 2005.

## 3. Results

### 3.1. Recapture and Survival Probability

Number of marked individuals in terrestrial habitat near the stream totaled 942 (462 males and 480 females). Sex ratio was not significantly different from unity, both when considering all individuals (χ^2^ = 0.34, *p* = 0.55) or when considering only individuals that were recaptured at least once (χ^2^ = 0.03, *p* = 0.84). Percentage of individuals that were recaptured at least once was 47.13% (48.5% in males and 45.8% in females).

**Figure 2:**
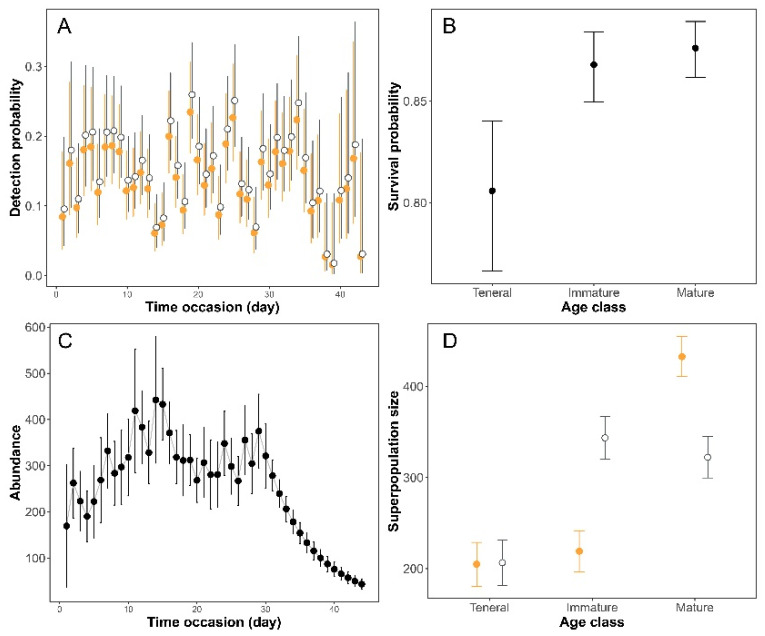
Predictions of the Cormack–Jolly–Seber model and POPAN model for capture–mark–recapture of *Platycnemis subdilatata* in northeastern Algeria. (**A**) Detection probability of males (grey open circle) and females (orange solid circle) across time occasion. (**B**) Survival probability of teneral, immature, and mature individuals. (**C**) Seasonal pattern of abundance estimated with the POPAN model. (**D**) “Superpopulation” size for teneral, immature, and mature individuals (males are in grey open circle and females are in orange solid circle).

The three tests of the goodness-of-fit were not significant (Table S3), which shows that there is no departure from the assumptions for the application of the CJS model. Estimated c-hat was 1.30, subsequently used to adjust model selection. Model selection for detection probability showed that the two best models (ΔAICc < 2) included the additive effects of time and sex and time alone (Table S4). There was a fluctuation in recapture probability across days that affected both sexes similarly, while males (0.16 [95%CI: 0.14–0.17]) showed a slightly higher recapture rate than females (0.14 [0.13–0.16]) (Figure 2A). Model selection for survival probability based on the best detection model (Sex + time) showed two top models (ΔAICc < 2), of which the most parsimonious included only age class in survival probability (Figure 2B) (Table S5). The survival model predicts that the survival probability of tenerals (0.80 [0.77–0.84]) was smaller than that of immature (0.87 [0.85–0.88]) and mature individuals (0.88 [0.86–0.89]). Based on the average survival probability (0.86 [0.85–0.87], we estimated an average longevity of 6.8 (95%CI: 6.27–7.39) days, but our observed maximum longevity was 48 days for males and 37 days for females.

We used POPAN models to estimate the population size of *P. subdilatata* in the terrestrial habitat using the best CJS models for survival and detection. Model selection showed that the best model for probability of entry included time occasion and sex, revealing that entry varied between males and females and from one occasion to another (Table S6). Number of individuals showed a seasonal pattern with a peak in the middle of the flight season (Figure 2C). We used the POPAN model to estimate a superpopulation size that varied among age classes. The largest superpopulation size was for mature individuals, and the lowest for tenerals (Figure 2D). By excluding the effect of age class and sex [Ψ (.)*p*(~time)*pent*(~time)*N*(.)], the superpopulation size was estimated to 1745 (1636–1861) individuals.

### 3.2. Occupancy of Terrestrial Habitat

**Figure 3:**
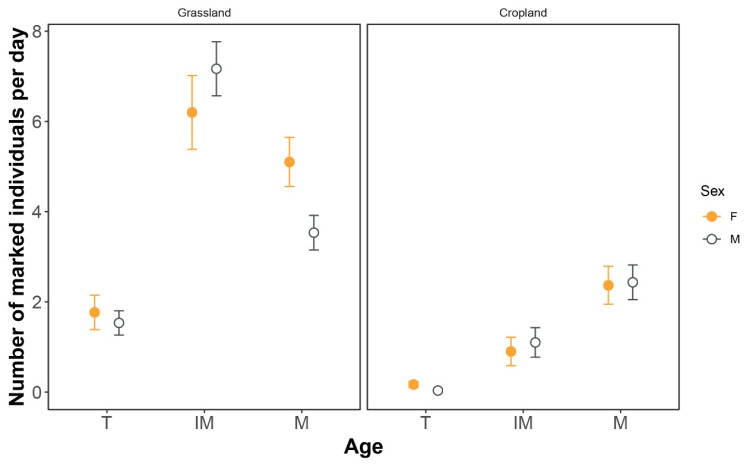
Average number of marked individuals per day of *Platycnemis subdilatata* across three age classes and sexes in grassland and cropland in northeastern Algeria. Grassland (3–5 m away from the water) was closer to the water than cropland. Error bars are 95% confidence intervals. T: teneral; IM: immature; M: mature individuals.

Based on 942 marked individuals in both grassland and cropland near the Boumahra stream, the spatial distribution of *P. subdilatata* in these habitats near the stream is shown in Figure 3. Number of individuals recorded per day was larger in grassland than in cropland (Table 1). Spatial distribution of males and females was similar in both grassland and cropland, revealed by the non-significant effect and interactions of sex with land type and age. Age had a significant main effect and interacted with land type (Table 1). Number of tenerals was lower than for immature and mature individuals in both grassland and cropland. Immature individuals showed a disproportionately higher number in grassland compared to other age classes. Number of mature individuals was higher than immature individuals in cropland.

### 3.3. Agricultural Extent and Occupancy

**Figure 4:**
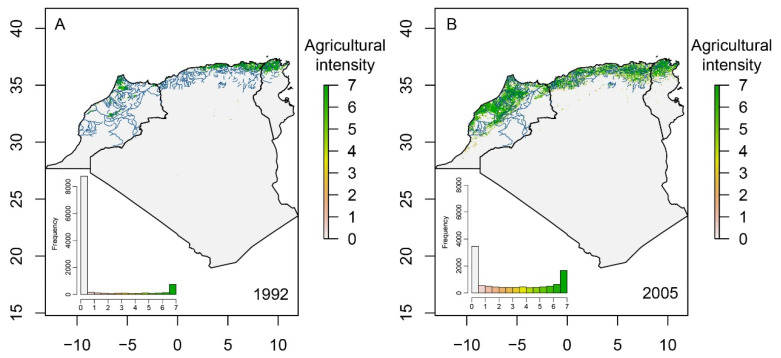
Change in the extent and intensity of the agriculture in North Africa from 1992 (**A**) to 2005 (**B**). The agricultural intensity ranges from 0 to 7 where 0 indicates the absence of agricultural activity and 7 indicates intense practices [47]. The main watercourses of the region are displayed in blue. The histogram shows the frequency distribution of agricultural intensity around the main watercourses (2 km buffer). Note the abrupt decline in the frequency of low agricultural intensity and the increase in the frequency of the other intensities from 1992 to 2005.

The agricultural land cover in North Africa increased by 321%, from 57,192 km^2^ to 241,010 km^2^ between 1992 and 2005 (Figure 4). The highest agricultural expansion was recorded in Morocco with an increase of 573%, then Algeria with 296%, and Tunisia with 80%. In addition, the intensity of agriculture was higher in 2005 than 1992 (Wilcoxon test: *p* < 0.001). For instance, intense practices (index values = 6–7) increased from 26.7% of all agricultural areas in 1992 to 45.8% in 2005. By limiting the area to the main rivers of the region and calculating the average intensity of agriculture within 4 km^2^-hexagons around the watercourse in 1992 and 2005, we found that the number of hexagons unoccupied by agriculture declined from 79.5% in 1992 to 26.2% in 2005, and the average intensity of agriculture increased by 239%, from 0.86 ± 2.07 (median = 0) in 1992 to 2.94 ± 2.66 (median = 2.49) in 2005 (Figure 4).

### 3.4. Distribution Modelling

The analysis of our compilation of studies resulted in 1284 records of presence–absence for the studied species (Table S1). After excluding points outside the expected range (Figure S1), we obtained 1070 records. *Platycnemis subdilatata* was present in 187 (17.5% of all records). All SDMs algorithms had relatively good predictive accuracies in terms of AUC and TSS (Table S7). RF, MAXENT, BRT, and GAM had AUC > 0.8, and the rest of the models had AUC ranging between 0.70 and 0.79. The highest TSS was recorded in RF (0.55), MAXENT (0.52), and BRT (0.50), and the rest of the models had values ranging between 0.36 and 0.49, with the lowest value recorded in BIOCLIM.

**Figure 5:**
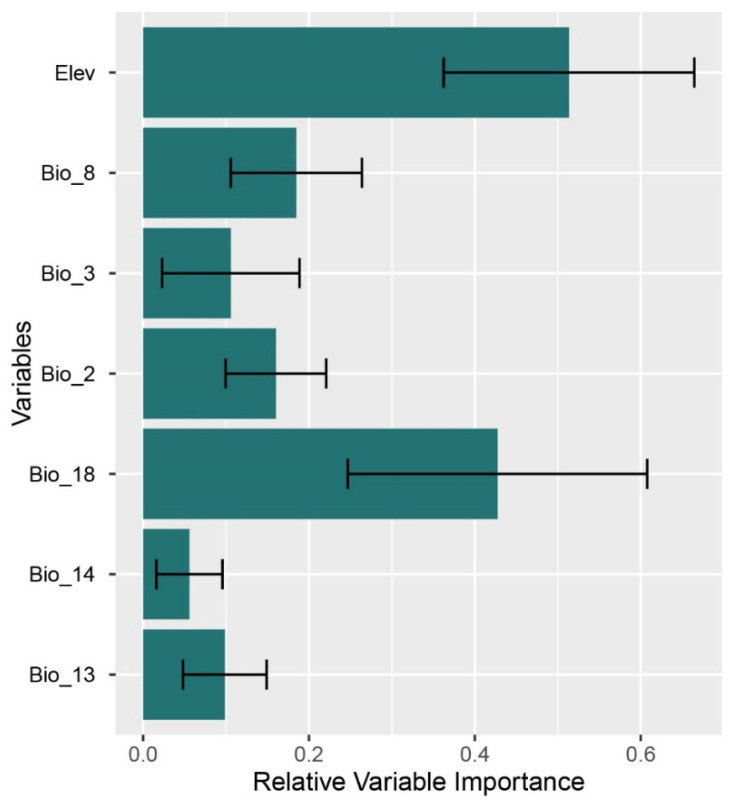
Relative variable importance for the species distribution models of *Platycnemis subdilatata* in North Africa based on 11 different algorithms and the test-dependent dataset. Bio2: mean diurnal range, Bio3: isothermality, BIO8: mean temperature of wettest quarter, Bio13: precipitation of wettest month, Bio14: precipitation of driest month, Bio18: precipitation of warmest quarter, Elev: elevation. More details in Table S2.

**Figure 6:**
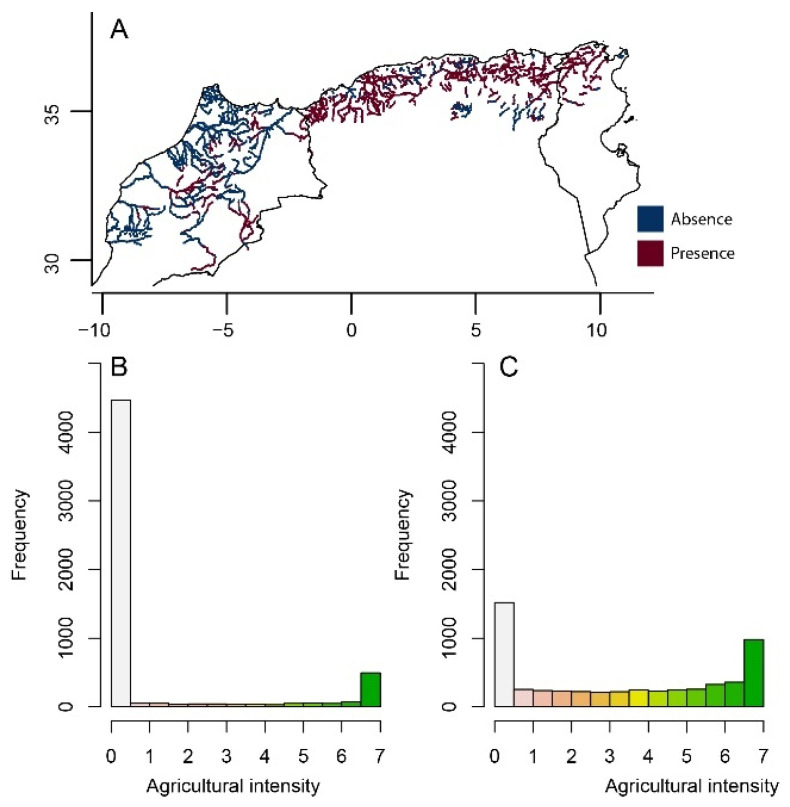
Predicted geographic range of *Platycnemis subdilatata* and the intensity of agriculture in North African lotic habitats. (**A**) Geographic range of *P. subdilatata* derived from species distribution modelling restricted to the main North African rivers, based on the ensemble model of 11 algorithms. The frequency distribution of agricultural intensity in 1992 (**B**) and 2005 (**C**) around (2 km buffer) the portion of the watercourse where the species lives. Note the abrupt decline in the frequency of low agricultural intensity and the increase in the frequency of the other intensities from 1992 to 2005.

There was some variability in the variable importance across models. Elevation and Bio18 (precipitation of warmest quarter) had the highest predictive power across all methods (relative importance > 0.20) (Figure 5). After evaluating the ensemble model, we estimated a threshold of occurrence of the species of 0.26, which was used to generate the presence–absence distribution restricted to the main northern rivers where the species potentially occurs (Figure 6). In the predicted species range, the occurrence of agriculture increased from 21.4% in 1992 to 78.1% in 2005 of the total area. The agricultural intensity of the two years was significantly different (Wilcoxon test: *p* < 0.001) with an average ± SD of 1.0 ± 2.24 (median = 0) in 1992 and 3.26 ± 2.65 (median = 3.21) in 2005 (226% increase).

## 4. Discussion

We showed that there has been a substantial increase in frequency of croplands and intensity of agricultural activity near the riparian zone of North African lotic habitats. We confirmed that the population size of *P. subdilatata* in terrestrial habitats near aquatic reproductive sites was large, and the damselfly used the terrestrial habitats, including grasslands and croplands near water, regularly. This is likely one reason why this species has survived well, whereas many other species in the same area such as *Calopteryx exul* have declined [48].

### 4.1. Demographic Parameters

Unlike many other studies that assessed sex ratio in reproductive sites where males were more frequent than females [49], there was a non-significant deviation from the equilibrium in the number of marked males and females in the terrestrial habitat. Sex ratio at emergence, which was assessed in the same site and during the same year, was not significantly different from 1:1 [26]. The typical male-biased sex ratio at reproductive sites (aquatic habitat) is believed to be the result of habitat segregation [49], with females occupying terrestrial habitats as an adaptation to escape male harassment. Absence of this pattern here suggests that, in the early morning, both sexes forage at the same time before they start mating.

Our CMR survey was carried out in a terrestrial habitat near the watercourse, which is different than most other studies on odonates in Northern Africa [50,51,52], where marking and recapture are conducted across the bank vegetation. Here, recapture probability was low, likely due to individual movement outside the sampling area, probably to change foraging sites [53]. Recapture probability was similar in males and females, which suggests that both sexes use similar foraging sites. The survival probability of *P. subdilatata* was not influenced by sex, suggesting that, despite the sexual color dimorphism, both sexes have a similar probability of being predated. There are three mutually non-exclusive explanations of the similarity in survival probability in both sexes. First, as the species is non-territorial, one would expect the absence of the costs related to male–male contests, which often lead to male-biased mortality [54]. Second, using the same foraging sites exposes both sexes to similar terrestrial predators. Third, both sexes spend the entire oviposition period linked together (contact-guarding), which also homogenizes the predation risks from aquatic and aerial predators. Age affected the survival probability. Tenerals showed lower survival than immature and mature individuals. Despite the careful manipulation of individuals during marking (we let the individual dry before marking), it is likely that the handling affected the survival of newly emerged individuals [55] due to their soft, fragile wings and body. However, the handling effect was probably also associated with a potential increased mortality risk for this age class due to its reduced ability to move and its slow flight, which makes tenerals more vulnerable to predation (e.g., robber flies and jumping spiders) [17]. Although immature and mature individuals showed similar survival probabilities, survival typically declines with age, most likely due to senescence [56]. Nevertheless, the conducted CMR encompassed inevitable challenges. For instance, regardless of the daily sampling visits, it was not possible to control the dispersal of individuals outside the sampling area. This might have reduced the recapture probability and underestimated lifespan.

### 4.2. Population Size

Number of marked adults was twice as high as the number of exuviae collected in a previous study at the same site and the same year [26]. Considering that much of the stream was shaded, this might suggest that the marked individuals came from the nearby stream and elsewhere. Our CMR sampling yielded an estimated population size of about 1750 adults observed in terrestrial habitat near the stream, suggesting that a large number of individuals (if not the entire population) used the terrestrial areas in the vicinity of the reproductive sites to forage and rest [17]. Our estimated population size varied across age classes and sex. Notably, the lower numbers of immature females compared to immature males suggested that they occupy a different habitat, whereas the higher numbers of mature females compared to mature males suggested that the latter stay near the water more often. Since the areas near the water were converted to agricultural land, which is usually the case in North Africa, *P. subdilatata* and most lotic odonates must have experienced a shift in habitat use from natural mixed plant communities to homogenous croplands. In addition, the long flight season of the species (from April to September) indicates that it occupies terrestrial habitats for much of the year (spring, summer, and early autumn) despite agricultural activities (mowing, irrigation, and pesticide application) being intense at these times.

### 4.3. Terrestrial Habitat Occupancy

We found that *P. subdilatata* occupied not only natural grassland but also cropland near the stream, although with fewer individuals in cropland compared to grassland. This could be for two reasons. The focal cropland was farther from the water than the grassland, and local damselflies, as opposed to dragonflies, usually stay closer to the water [57], structuring the population in space (population density decays with the distance from the water). Additionally, cropland might be less attractive than grassland due to the potential difference in prey diversity and abundance [58,59]. The observed age-dependent spatial structure means that while tenerals stay near water, mature adults occupy areas farther from the water, typical of odonates [17]. Right after emergence, odonates make a maiden flight, which is often short in damselflies. During the first day of the adult life, tenerals limit their movement and expose themselves to the sun so as to harden their body and wings. Immature individuals are more mobile as they have harder morphological structures and need to forage to build mass and reach maturity. Mature individuals also need to fly farther from the water to forage or, in the case of males, occasionally search for mates [60].

### 4.4. Geographic Distribution

The increased Odonata surveys in North Africa in recent years, particularly in lotic habitats [25,52,61,62], allowed us to carry out spatial analysis of the distribution of the widespread *P. subdilatata* in the region, and to test for the extent of prevalence of agroecosystems associated with lotic habitats. Our SDM showed that (1) *P. subdilatata* has a wide distribution in North Africa, (2) the estimated geographic overlap between the species range and agriculture was 21.4% in 1992, and 78.1% in 2005, and (3) the intensity of agricultural practices increased by 226%. This substantial increase is due to political strategies to meet food security and economic development, especially in keeping with the rapid human population growth. Agricultural expansion and intensification have multiple implications for the structure and abundance of lotic assemblages. First, rivers and streams are extensively used for irrigation, which not only reduces the physical space for aquatic fauna but possibly, through the activity of extraction, sucks out aquatic stage. Second, regular use of pesticides and fertilizers leads to contamination and eutrophication of the water with substantial implications for the lotic fauna [63], as well as direct and indirect exposure to toxicity in the terrestrial habitat where odonates forage and rest [64]. Although no effects have been observed for the species studied, environmental modifications towards agricultural use could be greatly affecting odonates such as *Calopteryx exul*, *Gomphus lucasii*, *Onychogomphus boudoti* (Ferreira et al. [22]), which often coexist with *P. subdilatata* [53,65].

Compared to other endemic lotic species in North Africa [28], *P. subdilatata* has a substantially larger distribution. Occurrence of the species in the river downstream, where pollution is the highest within the entire watershed [66], shows that it is not highly pollution-sensitive [67], unlike other local lotic species [48,51]. Presence of the species across a relatively large elevation range also indicates a wide thermal tolerance and potential plasticity in life history [68]. Such ecological traits have probably enabled *P. subdilatata* to maintain a wide distribution and resist environmental changes, such as fluctuations in the hydrological regime, which are challenging for other sympatric species [48]. Additionally, considering the current conservation status of the species (least xoncern on the IUCN Red List) and the stable population levels, the species showed a considerable level of resilience in the face of agriculturally-driven habitat fragmentation and disturbance.

Given global climate change and the dynamics of the species’ geographic range, it is interesting to understand what limits the geographic boundaries of the species to North Africa and estimate the likelihood of range expansion to the north (particularly from Morocco to Spain) as observed in other odonates during the recent years [69,70].

## 5. Conclusions

We investigated how lotic odonate habitats are associated with agriculture in North Africa, a research avenue that has received little attention in odonate conservation. We found the lotic odonate habitat often includes agricultural lands, which are frequently used by adults. Our knowledge of the impact of simplified monoculture agroecosystems on prey type, abundance, and quality, as well as on the behavior of lotic fauna is still extremely limited. Dominance of agricultural activity near lotic habitats is most likely a global phenomenon, but solutions to reduce its impacts on lotic biodiversity are still lacking in northern Africa. Lind et al. [71] suggest that a >30 m buffer is necessary to reach effective ecological functioning of the riparian zones. The current study highlights that, exceptionally, some species such as *P. subdilatata* could maintain large populations in a heavily modified lotic environment, but this is most likely not the case for the majority of the lotic odonate assemblages in the area. In addition, the apparent resilience of the species could hide an existing extinction debt that would eventually manifest in the future [72]. Further studies should be conducted at the assemblage scale to obtain a more general view of the impact of agricultural expansion on lotic odonates in North Africa.

## Figures and Tables

**Table 1:** Summary statistics of the negative binomial model assessing the effect of age class, land-type and sex on the number of marked adult individuals of *Platycnemis subdilatata* per day. Baseline level for contrast calculation for age was immature individuals (IM), for sex was female, and for land was cropland.

	Estimate	Std. Error	*z*	*p*
(Intercept)	−0.105	0.214	−0.491	0.6233
Age[M]	0.967	0.263	3.679	0.0002
Age[T]	−1.686	0.505	−3.340	0.0008
Sex[Male]	0.201	0.292	0.687	0.4920
Land[Grassland]	1.930	0.246	7.856	<0.0001
Age[M] × Sex[Male]	−0.173	0.362	−0.478	0.6329
Age[T] × Sex[Male]	−1.810	1.142	−1.586	0.1128
Age[M] × Land[Grassland]	−1.162	0.314	−3.695	0.0002
Age[T] × Land[Grassland]	0.431	0.545	0.791	0.4292
Sex[Male] × Land[Grassland]	−0.056	0.337	−0.166	0.8683
Age[M] × Sex[Male] × Land[Grassland]	−0.339	0.439	−0.772	0.4402
Age[T] × Sex[Male] × Land[Grassland]	1.524	1.179	1.292	0.1962

T: tenerals; M: mature individuals.

## Data Availability

Data supporting reported results are accessible here: https://github.com/rassimkhelifa/Platycnemis_2021 (accessed on 2 March 2021).

## Supplementary Materials

The following are available online at https://www.mdpi.com/article/10.3390/insects12040353/s1, Figure S1: Overall climatic conditions of the North African region where *Platycnemis subdilatata* occurs., Figure S2: ROC-AUC value of the eleven SDM models with the subsampling replication, Table S1: List of studies and database used to generate presence–absence data for *Platycnemis subdilatata* in Northern African region (Algeria, Tunisia, and Morocco), Table S2: WorldClim temperature and precipitation variables used to build the species distribution models, Table S3: Results of the goodness-of-fit of the multistate model for capture–mark–recapture data of *Platycnemis subdilatata*, Table S4: Model selection of the capture–mark–recapture Cormack–Jolly–Seber model for recapture probability, Table S5: Model selection of the capture–mark–recapture Cormack–Jolly–Seber model for survival probability, Table S6: Model selection of the capture–mark–recapture POPAN model, Table S7: Species distribution model mean performance using test dataset (generated using partitioning).
