# Supplementary material for: Remarkable Population Resilience in a North African Endemic Damselfly in the Face of Rapid Agricultural Transformation
Source: Insects. 2021 Apr 15;12(4):353. doi: 10.3390/insects12040353 (PMC8071254; doi:10.3390/insects12040353)
Supplement: Supplementary file 1 [file insects-12-00353-s001.zip › insects-1160777-supplementary.pdf]

Supplementary material

Remarkable population resilience in a North African endemic damselfly in the face of rapid agricultural transformation

Rassim Khelifa[1,2*], Hayat Mahdjoub[2], Affef Baaloudj[3], Robert A. Cannings[4] and Michael J. Samways[5]

[1] Department of Zoology, University of British Columbia, Vancouver, Canada, BC, V6T 1Z4

[2] Biodiversity Research Centre, University of British Columbia, Vancouver, Canada, BC, V6T 1Z4

[3] Laboratory LBEE: Biology, Water and Environment, Faculty SNV-STU, University 8 May 1945 Guelma, BP 401 24000, Guelma, Algeria

[4] Entomology, Royal British Columbia Museum, 675 Belleville Street, Victoria, B.C., Canada V8W 9W2

[5] Department of Conservation Ecology and Entomology, Stellenbosch University, Victoria Street 7602, Stellenbosch, South Africa

*Corresponding author. Email: rassimkhelifa@gmail.com

Running title: Agriculture and odonates

Supplementary figures

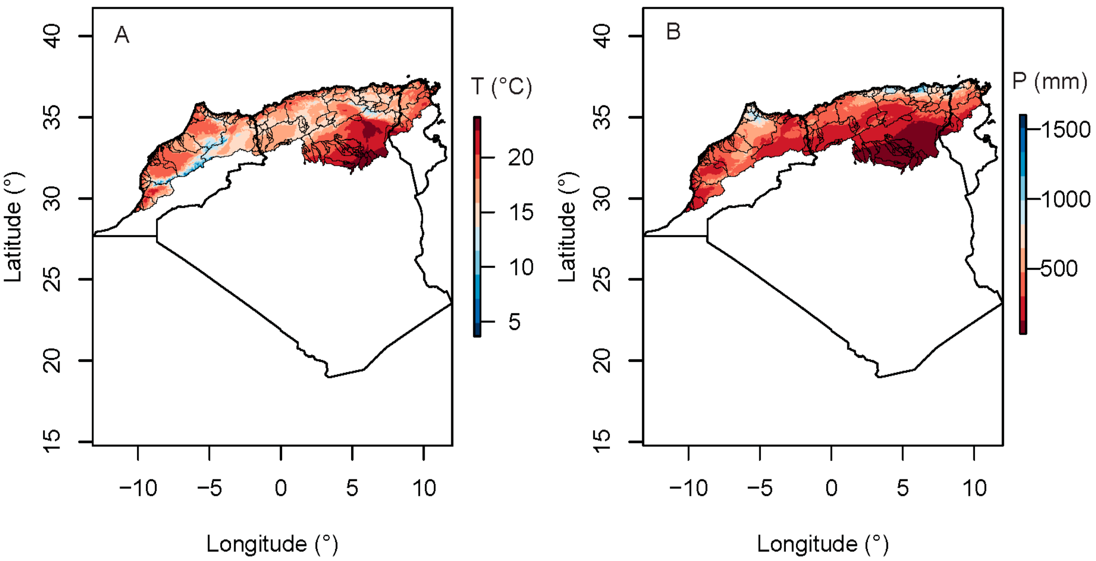

Figure S1. Overall climatic conditions of the North African region where *Platycnemis subdilatata* occurs. A. Annual average temperature (T). B. Annual precipitation (P). The outlines are watershed basins obtained from [69]. Climate data were based on WorldClim2.1[26] for the period 1980-2018.

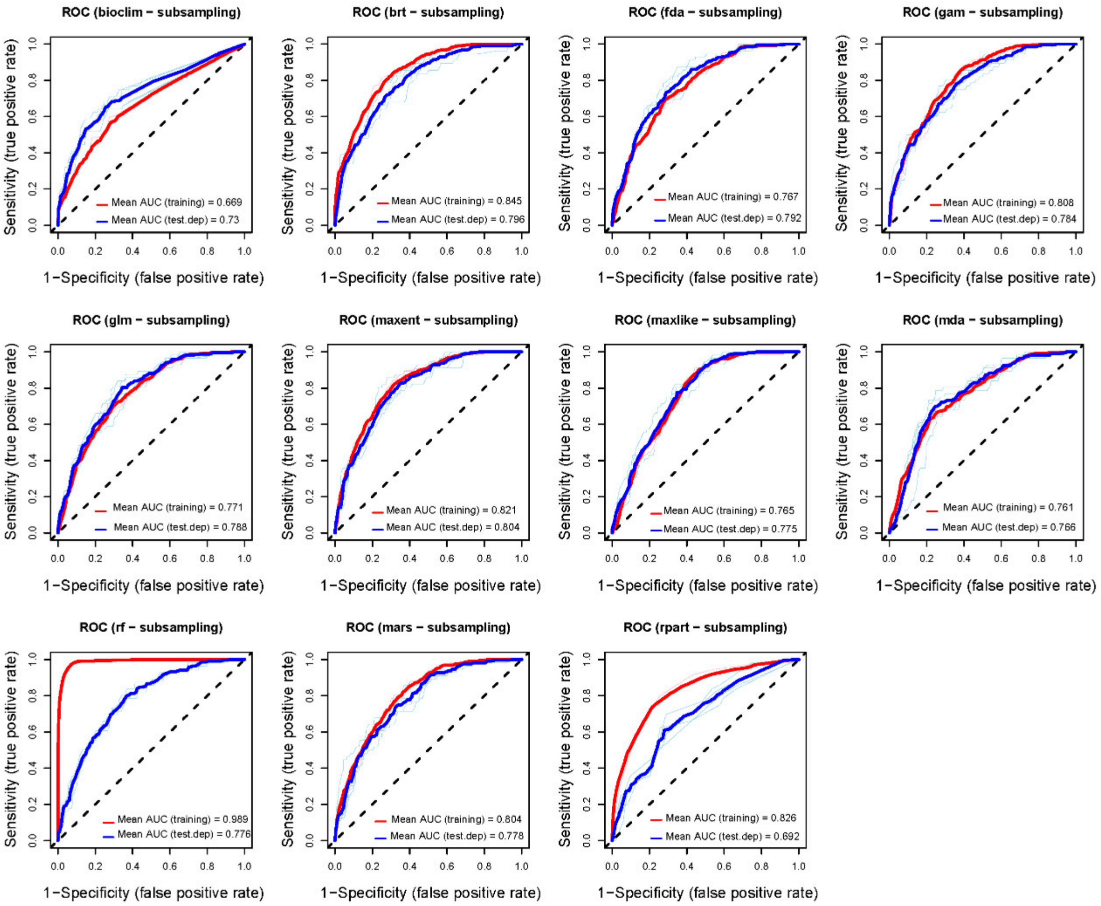

Figure S2. ROC-AUC value of the eleven SDM model with the subsampling replication.

Supplementary tables

Table S1. List of studies and database used to generate presence-absence data for *Platycnemis subdilatata* in Northern African region (Algeria, Tunisia, and Morocco).

Country	Reference
**Algeria**	Bouchelouche, D., Kherbouche-Abrous, O., Mebarki, M., Arab, A., & Samraoui, B. (2015). The Odonata of Wadi Isser (Kabylia, Algeria): Status and environmental determinants of their distribution. *Revue d'Ecologie* 70, 248-260.
	Chelli, A. M., & Moulaï, R. Diversity and ecological diagnosis of dragonflies of high-mountain temporary ponds in the Akfadou massif forest (Algeria). Zoology and Ecology 29(1), 28-37
	Chelli, A., & Moulaï, R. (2019). Ecological characterization of the odonatofauna in lotic and lentic waters of northeast Algeria. *Annales de la Société Entomologique de France (NS)* 55, 430-445
	Demnati, F., Allache, F., & Cohez, D. (2019). Contribution A La Connaissance De L'odonatofaune Du Bassin Du Chott Melghir (Algérie). *Bull. Soc. Zool. Fr*, *144*(2), 95-104.
	Yalles Satha, A., & Samraoui, B. (2017). Environmental factors influencing Odonata communities of three mediterranean rivers: Kebir-east, Seybouse, and Rhumel wadis, northeastern Algeria. *Revue d'Ecologie*72: 314-329.
	Senouci, H., & Bounaceur, F. (2018). Contribution to the study of diversity and abundance of odonates in some wet biotopes in Tiaret region, Algeria. *Plant Archives*, *18*(1), 555-560.
	Khelifa, R. (2019). Sensitivity of biodiversity indices to life history stage, habitat type and landscape in Odonata community. *Biological Conservation*, *237*, 63-69.
	Khelifa, R., Mellal, M. K., Zouaimia, A., Amari, H., Zebsa, R., Bensouilah, S., ... & Houhamdi, M. (2016). On the restoration of the last relict population of a dragonfly Urothemis edwardsii Selys (Libellulidae: Odonata) in the Mediterranean. *Journal of Insect Conservation*, *20*(5), 797-805.
**Morocco**	El Haissoufi, M., Bennas, N., El Mohdi, O., & Millán, A. (2010). Analyse préliminaire de la vulnérabilité des Odonates (Odonata) du Rif Occidental (Nord du Maroc). *Boletín de la Sociedad entomológica Aragonesa*, *46*, 345-354.

	El Haissoufi, M., Lmohdi, O., Bennas, N., Mellado, A., & Millan, A. (2008). Les Odonates du bassin versant Laou (Rif occidental, Maroc). *Trav. Instit. Scient. Rabat*, 5, 47-59.
	Juillerat, L., & Monnerat, C. (2009). Odonata in southern Morocco, with first records of Orthetrum ransonnetii and Sympetrum sinaiticum (Odonata: Libellulidae). *Libellula*, *28*, 97-115.
	Taybi, A. F., Mabrouki, Y., Berrahou, A., Sbaa, M., & Brochard, C. (2019). New data on the dragonfly fauna (Odonata) of the Moulouya River Basin and the Oriental Region, Morocco. *Arxius de Miscel· lània Zoològica*, *17*, 85-108.
**Tunisia**	Korbaa, M., Ferreras-Romero, M., Ruiz-García, A., & Boumaiza, M. (2018). TSOI− a new index based on Odonata populations to assess the conservation relevance of watercourses in Tunisia. *Odonatologica*, *47*(1/2), 43-72.
	Sellami, E. L., Meurgey, F., Barbouche, N., & Romdhane, M. S. (2015). Odonates dans les principaux cours d'eau du parc national de l'Ichkeul (Tunisie). *Entomologie faunistique-Faunistic Entomology* 68, 93-100
	Jödicke, R., Arlt, J., Kunz, B., Lopau, W., & Seidenbusch, R. (2000). The Odonata of Tunisia. *International Journal of Odonatology*, *3*(1), 41-71.
**All three countries**	GBIF.org (25 February 2021). GBIF Occurrence Download https://doi.org/10.15468/dl.c2rc2d.

Table S2. WorldClim temperature and precipitation variables used to build the species distribution models. To reduce collinearity among variables, only the listed seven variables were retained (BIO2, BIO3, BIO8, BIO13, BIO14, BIO18, elevation) after applying a threshold of Variance Inflation Factor (VIF) of 10.

**Variable**	**Description**
**BIO2**	Mean Diurnal Range (Mean of monthly (max temp - min temp))
**BIO3**	Isothermality (BIO2/BIO7) (* 100)
**BIO8**	Mean Temperature of Wettest Quarter
**BIO13**	Precipitation of Wettest Month
**BIO14**	Precipitation of Driest Month
**BIO18**	Precipitation of Warmest Quarter
**Elev**	Elevation

Table S3. Results of the Goodness-of-fit of the multistate model for capture-mark-recapture data of *Platycnemis subdilatata*. Test2 check for equality in the chances of capture throughout occasions, whereas Test3 look for homogeneity of survival probability over time. The non-significance of the tests shows that the assumptions of the CJS model are fulfilled.

**Test**	χ^2	**df**	**P value**
**TEST2**	157.24	217	0.9992
**TEST3**	91.06	231	1.0000
**Total**	248.30	448	1.0000

Table S4. Model selection of the capture-mark-recapture Cormack-Jolly-Seber model for recapture probability. Survival probability was fixed to one. Time (capital T) is the continuous time occasion, and time is the categorical time occasion.

Model	npar	QAICc	ΔQAICc	weight	QDeviance
Ψ(.)p(time)	44	4776.0	0.00	0.556	2932.1
Ψ(.)p(Sex + time)	45	4776.5	0.46	0.440	2930.5
Ψ(.)p(.)	2	4787.8	11.80	0.002	3030.1
Ψ(.)p(Sex)	3	4788.4	12.37	0.001	3028.7
Ψ(.)p(Time)	3	4789.8	13.79	0.001	3030.1
Ψ(.)p(Sex + Time)	4	4790.3	14.30	0.000	3028.6
Ψ(.)p(Sex × Time)	5	4791.2	15.22	0.000	3027.5
Ψ(.)p(Sex × time)	87	4823.0	46.95	0.000	2886.4

Ψ: Survival probability; p: recapture probability

Table S5. Model selection of the capture-mark-recapture Cormack-Jolly-Seber model for survival probability. Time (capital T) is the continuous-time occasion, and time is the categorical time occasion. Age has three classes: teneral, immature, and mature individuals.

**Model**	**npar**	**QAICc**	**ΔQAICc**	**weight**	**QDeviance**
Ψ(Age)p(Sex + time)	47	4768.5	0.00	0.645	2918.3
Ψ(Age + Sex)p(Sex + time)	48	4770.4	1.88	0.252	2918.1
Ψ(Age × Sex)p(Sex + time)	50	4772.9	4.41	0.071	2916.4
Ψ(.)p(time)	44	4776.0	7.48	0.015	2932.1
Ψ(.)p(Sex + time)	45	4776.5	7.94	0.012	2930.5
Ψ(Sex)p(Sex + time)	46	4778.3	9.79	0.005	2930.2
Ψ(.)p(.)	2	4787.8	19.27	0.000	3030.1
Ψ(.)p(Sex)	3	4788.4	19.85	0.000	3028.7
Ψ(.)p(Time)	3	4789.8	21.27	0.000	3030.1
Ψ(.)p(Sex + Time)	4	4790.3	21.78	0.000	3028.6
Ψ(.)p(Sex × Time)	5	4791.2	22.69	0.000	3027.5
Ψ(.)p(Sex × time)	87	4823.0	54.43	0.000	2886.4

Ψ: Survival probability; p: recapture probability

Table S6. Model selection of the capture-mark-recapture POPAN model.

Model	npar	QAICc	ΔQAICc	weight	QDeviance
Ψ(Age)p(time + Sex)pent(time +Sex)N(Sex)	70	5589.0	0.00	0.96	-941.3
Ψ(Age)p(time + Sex)pent(time)N(Sex)	67	5595.5	6.51	0.04	-928.3
Ψ(Age)p(time + Sex)pent(.)N(Sex)	50	5754.9	165.83	0.00	-732.7

Ψ: Survival probability; p: recapture probability; pent: probability of entry; N: Superpopulation size

Table S7. Species distribution model mean performance using test dataset (generated using partitioning)

Methods	Full name	AUC	COR	TSS	Deviance
RF	Random Forests	0.83	0.51	0.55	0.73
MAXENT	Maximum Entropy	0.82	0.43	0.52	1.04
BRT	Boosted Regression Trees	0.81	0.45	0.50	0.80
GAM	Generalized Additive Models	0.80	0.42	0.49	0.85
FDA	Flexible Discriminant Analysis	0.79	0.41	0.47	0.79
GLM	Generalized Linear Models	0.79	0.40	0.48	0.78
MARS	Multivariate Adaptive Regression Splines	0.79	0.40	0.47	0.81
MAXLIKE	Likelihood-Based Estimator Adopted for Presence-Only Data	0.77	0.36	0.46	2.43
MDA	Multiple Discriminant Analysis	0.76	0.36	0.47	1.05
RPART	Recursive Partitioning and Regression Trees	0.73	0.37	0.41	0.91
BIOCLIM	BIOCLIM	0.70	0.31	0.36	1.44
